# Development of Knitted Strain Sensor Optimized for Dumbbell Exercise and Evaluation of Its Electrical Characteristics

**DOI:** 10.3390/s25123685

**Published:** 2025-06-12

**Authors:** Hee-Ji Choi, Youn-Hee Kim

**Affiliations:** Department of Convergence Design and Technology, Kookmin University, Seoul 02707, Republic of Korea; hichio1228@kookmin.ac.kr

**Keywords:** knitted strain sensor, conductive yarn, smart textile, bending test, dumbbell

## Abstract

With growing interest in wearable technologies, the development of flexible sensors and products that can monitor the human body while being comfortable to wear is gaining momentum. While various textile-based strain sensors have been proposed, their implementation in practical, exercise-specific applications remains limited. In this study, we developed a knitted strain sensor that monitors elbow angles, focusing on dumbbell exercise, which is a basic exercise in sports, and verified its performance. The material of the developed knitted strain sensor with a plain stitch structure comprised a silver-coated nylon conductive yarn and an acrylic/wool blended yarn. To evaluate the electrical and physical characteristics of the developed sensor, a textile folding tester was used to conduct 100 repeated bending experiments at three angles of 30°, 60°, 90° and speeds of 10, 30, 60 cpm. The system demonstrated excellent elasticity, high sensitivity (gauge factor = 698), fast responsiveness, and reliable performance under repeated stress, indicating its potential for integration into wearable fitness or rehabilitation platforms.

## 1. Introduction

As research into wearable electronic devices that detect human body movements and monitor respiration or heart rate becomes more active, interest in embedding lightweight, comfortable, and flexible sensors into textiles is increasing [1]. Among wearable electronic devices, strain sensors have been investigated in various application fields because they can recognize and distinguish signals from the human body and the mobility of related organs [2,3,4,5,6]. In particular, knitted strain sensors are lightweight and flexible, making them comfortable to wear and suitable for joint monitoring [7]. Factors affecting the performance of sensors include fabric structure, stress, material, and size [8,9], and many studies are being conducted to develop sensors optimized for each use. For example, Tohidi et al. [10] developed a sensor with a plain stitch structure and investigated the effect of structural parameters of the fabric on the electromechanical properties of the sensor. Seyedin et al. [11] examined how different loop patterns affect the sensor’s detection mechanism. The detection performance of the sensor can be customized by changing the loop structure or inserting a stitch into the knit structure. Liu et al. [12] reported that the structure of the sensor has a great influence on the detection performance, and that the sensor with the plain stitch structure generally has the best detection performance and conductivity. Previous studies have reported the influence of the structural design of the knitted structure on the electromechanical performance of the sensor. In particular, the plain stitch structure showed the best performance overall. Meanwhile, studies verifying the real-world applicability of knitted strain sensors have also been reported. For example, Gupta et al. [13] developed a wearable knee brace with integrated knit sensors and evaluated the accuracy of the sensor by measuring the knee joint movements during walking and jogging activities. Warnke et al. [14] developed a knitted strain sensor for joint monitoring for medical motion capture and quantitatively analyzed its electromechanical properties to verify its performance. Sun et al. [15] developed a knitted vest with integrated sensors for shoulder motion monitoring and verified its detection performance in various motions. These efforts demonstrate the promising potential of knitted strain sensors in various wearable applications.

This study focuses on elbow joint movement in sports among various applications of knitted strain sensors. In many sports, such as baseball, tennis, and golf, postures such as elbow angles are important for improving performance and preventing injuries. To this end, players perform additional weight training to strengthen their arm muscles. A dumbbell is an exercise device that facilitates muscle growth through the simplest and most diverse form of exercise, and we want to evaluate the possibility of monitoring the elbow using a knitted strain sensor, focusing on the dumbbell exercise, which can be a basic exercise.

The important factors in dumbbell exercises are posture, movement speed, number of exercises, and duration of exercise. Accurate posture is important to strengthen the muscles in the target area, and the angle of the elbow is important because, during dumbbell exercises, the dumbbells are held by the hands. In addition, exercise should be performed slowly to provide sufficient muscle stimulation and promote faster growth [16].

For real-time monitoring, high sensitivity, excellent stability, a wide detection range, high elasticity, and rapid recovery from deformation are essential [17]. Sensitivity is an important factor in evaluating the performance of sensors, indicating changes in capacitance or electrical resistance to the rate of strain change caused by the mechanical stimulation of the sensor [18]. To measure bending and extension movements in real time during dumbbell exercises, the responsiveness of the strain sensor is important. Responsiveness can be verified by the speed at which the sensor responds to the bending movement and the time required to return to the extension movement. Dumbbell exercises are long-term, continuous exercises; thus, sensor values must be measured stably and accurately even during long-term repeated bending. This can be confirmed by the reproducibility of the sensor, which is a performance indicator.

In this study, we developed a highly stretchable knitted strain sensor for real-time elbow joint monitoring, focusing on dumbbell exercise, which can be a basic exercise among sports fields, and evaluated the electrical and physical characteristics of the sensor. The developed sensor had high sensitivity and was able to stably detect long-term repeated sensing based on excellent reproducibility, and it showed fast responsiveness to deformation. In addition, the system integration of sensors, microcontroller unit (MCU), lead wires, and spring snap button provides measurement accuracy and fast response time, and is wireless, making it convenient to use. These wireless systems are capable of real-time elbow joint monitoring and demonstrate practical applicability in wearable applications.

## 2. Materials and Methods

### 2.1. Structure and Working Mechanism

Silver-coated nylon conductive yarns have been used in wearable applications, such as human body monitoring, because they exhibit high conductivity, low contact resistance [19], and superior uniformity compared to stainless steel-based conductive yarns [20]. In this study, the conductive yarn used for sensor development was a silver-coated nylon conductive yarn of Amann (Bonnigheim, Germany) with a resistance of <530 Ω/m. Although Amann’s specification states less than 530 Ω/m, we performed independent resistance measurements on five 1 m long samples of conductive yarn. This evaluation was performed to confirm the exact nature of the uncertainty evaluation. The result was a mean resistance of 590.4 Ω/m with a standard uncertainty of ±3.6 Ω/m. A silver-coated nylon conductive yarn with Aman’s <85 Ω/m resistance was used as the lead wire to connect the developed sensor to the MCU. Aman’s silver-coated nylon conductive yarn with resistance <85 Ω/m had a mean resistance of 70.6 Ω/m and a standard uncertainty of ±4.2 Ω/m. As a result, we confirmed the difference between the resistance value of the conductive yarn specified by the manufacturer and the resistance value measured in this study. In addition, the conductive yarns were of the same type, but there was a difference in the resistance value of each conductive yarn, a phenomenon reported in previous studies. Stavrakis et al. [21] examined Aman’s silver-coated nylon conductive yarn to analyze the electrical properties of commercially available conductive yarns, and similar to the findings of this study, and found a significant discrepancy between the manufacturer’s specified resistance values and the actual measurements. Differences in resistance values are common phenomena that can occur depending on the manufacturing process (uniformity of coating), environmental factors (temperature, humidity, oxidation, etc.), and measurement conditions [22,23,24]. However, these differences do not have a significant impact on the functionality of the sensor, and the measured values are well within the scope of the intended application.

**Figure 1:**
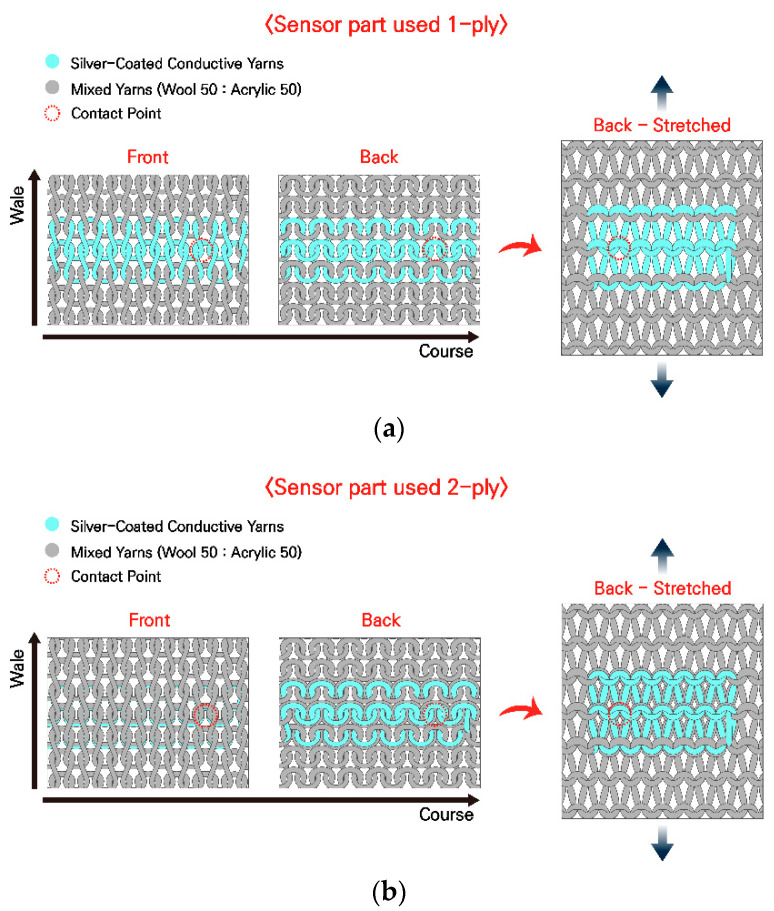
Knitted strain sensor. (**a**) schematic of 1-ply plain stitch; (**b**) schematic of 2-ply plain stitch.

In this study, the knitted strain sensor sample was manufactured using CMS330 KI TT SPORT E7.2 (STOLL, Rettingen, Germany) and a computerized flat knitting machine, and it was designed with a plain stitch structure. Figure 1a,b show diagrams of a knitted strain sensor with a plain stitch structure comprising both conductive and general yarns and the appearance of the sensor when pulled in the front, back, and wale directions. Figure 1a shows the most common plain stitch with the sensor part knitted with only 1-ply of a conductive yarn; thus, the conductive yarn appears on the front, back, and both sides. Figure 1b shows a plain stitch designed to use two needles on the sensor part to position 1-ply of a non-conductive yarn on the front and 1-ply of a conductive yarn on the back. Figure 1a,b show diagrams of a knitted strain sensor with a plain stitch structure comprising both conductive and non-conductive yarns and the appearance of the sensor when pulled in the front, back, and wale directions. Figure 1a shows the most common plain stitch structure with the sensor part knitted with only 1-ply of a conductive yarn; thus, the conductive yarn appears on the front, back, and both sides. Figure 1b shows a plain stitch structure designed to use two needles on the sensor part to position 1-ply of a non-conductive yarn on the front and 1-ply of a conductive yarn on the back. The sensor part of the sample comprised 2-ply, including 1-ply of a silver-coated conductive yarn and 1-ply of a non-conductive yarn from C&TEX (Seoul, Republic of Korea) at a 1:1 ratio of acrylic to wool, and the rest were made into a plain stitch structure with excellent elasticity using only 1-ply of a non-conductive yarn [25]. The overall length of the fabricated sample is 265 mm, the width is 90 mm, and the sensor part is 100 mm in length (88 wales) and 20 mm in width (14 courses). 

### 2.2. Measurement of Electromechanical Properties

**Figure 2:**
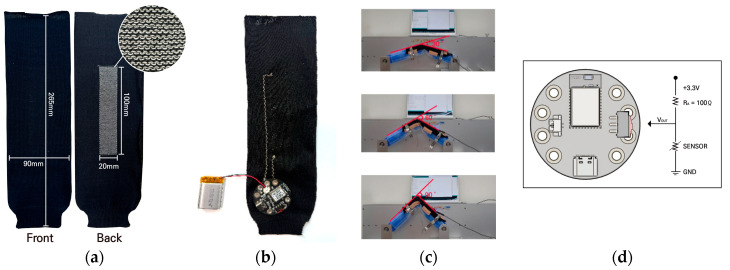
Bending experiment preparation: (**a**) knitted strain sensor sample (front, back); (**b**) knitted strain sensor with sewn lead wires; (**c**) image of folding tester bent at different angles; (**d**) schematic of voltage divider of sensor system.

Based on previous studies, we developed a knitted strain sensor with a 2-ply plain stitch structure (Figure 2a). The Arduino program (version 1.8.9) was used for data collection, and for this purpose, it was connected to the MCU by securing the spring snap button to the end of the lead wire (Figure 2b). The lead wire was sewn using a zigzag stitch, which is more durable than a straight stitch. This system integration is wireless, easy to use, and provides accurate measurements and fast responsiveness. The electrical and physical characteristics of the voltage and resistance changes according to the bending angle of the knitted strain sensor were investigated through repeated bending experiments using a textile folding tester, model CKFT-T400 (Netest, Hwasung-si, Republic of Korea) (Figure 2c). All knitted strain sensors require a pre-stretching process to maintain stable electrical characteristics; thus, each sample was pre-stretched five times before the experiment to ensure stable data measurement [26]. Dumbbell exercises are long-term and continuous; thus, correct posture and elbow angle are important [16]. Therefore, to confirm the possibility of motion recognition for elbow flexion angles and to examine the difference in resistance according to the angles, experiments were conducted at three angles: 30°, 60°, and 90°. The bending cycles for each experiment were 100 to confirm long-term repetitive bending performance. To determine changes in sensor resistance due to bending and extension, detailed measurements must be performed, including sensor values during bending and extension at low speeds. In addition, to sufficiently stimulate the muscles through dumbbell exercise, the speed of movement must be adjusted not too quickly [16]. Accordingly, the bending speed was tested at three speeds of 10, 30, 60 cycles per minute (cpm) based on positive and negative movements. The MCU used in the experiment was an Arduino-based ESP 32-PICO (Indifrog, Seongnam-si, Republic of Korea) (Figure 2d), which was connected to the Arduino program to output data every 0.1 s, and 100 repeated bending experiments were conducted.

## 3. Results and Discussion

### 3.1. Results of Repeated Bending Experiments

To confirm that the developed knitted strain sensor is capable of real-time monitoring of the elbow joint angle, its electrical and physical characteristics were evaluated in terms of sensitivity, responsiveness, and reproducibility. The voltage scale of the Arduino used in the experiment was 0–3.3 V, and the serial monitor measured the range of 0–4095. The ∆ sensor value extracted from the bending experiment was converted into an actual voltage value by substituting it into Equation (1) [27].

(1) $$Actual\;V_{B}=\frac{output\left(sensor\;value\right)\times 3.3}{4095}\;V_{A}=3.3-V_{B}[V]\;I_{A}=\frac{V_{A}}{R_{A}}\;R_{B}=\frac{V_{B}}{I_{A}}$$

In dumbbell exercises, the muscles used vary depending on the posture, and the right posture is important to prevent injuries caused by inaccurate posture; thus, the elbow joint angle should be accurately measured. Accordingly, the sensor must exhibit high sensitivity for measuring the elbow joint angle in various positions in detail. Similar to sensitivity, responsiveness is an important performance indicator of elbow joint monitoring during dumbbell exercise. To accurately recognize the posture and elbow joint angle in real time during exercise, the sensor must respond quickly when the arm is bent and quickly return to its initial state when the arm is extended; thus, repeatability is required as a performance indicator. To increase the effectiveness of dumbbell exercises, multiple repetitions and a long exercise period are required. For this purpose, the developed knitted strain sensor must exhibit reproducibility to reliably recognize continuous and repetitive movements.

In this study, GF and resistance change rate were measured at various bending angles and strains to evaluate the performance of the knitted strain sensor. Strain sensors based on metal materials generally have a cross-sectional area (A) that decreases as the length (R) increases (L+∆L) due to an external force, which increases the resistance. In contrast, as the length decreases, the resistance decreases, which is called the piezoresistive effect [28]. According to the principle of Equation (2), the resistance (R) of a metal with electrical conductivity p is determined by the cross-sectional area (A) and length (L); thus, the GF is calculated using Equation (3) when the initial resistance ($R_{0}$) changes according to the length change (∆L) [29].

(2) $$R=p\frac{L}{A}$$

(3) $$GF=\frac{\frac{∆R}{R_{0}}}{\frac{∆L}{L_{0}}}=\frac{∆R}{{\varepsilon R}_{0}}$$

**Figure 3:**
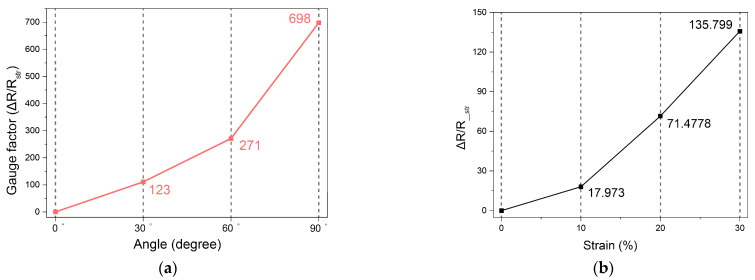

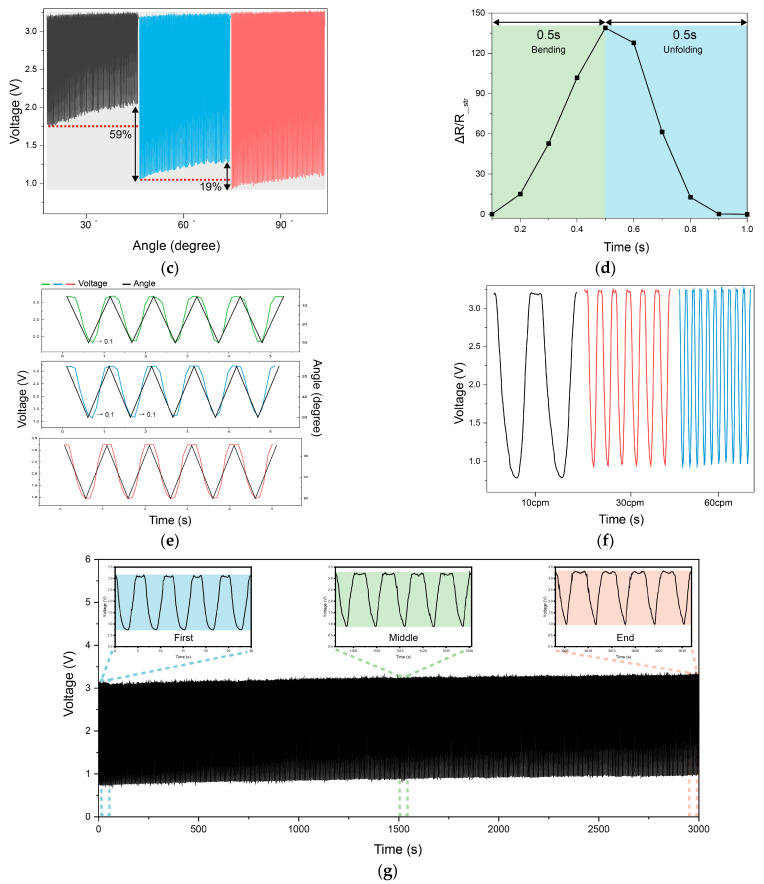
Results of repeated bending tests: (**a**) GF by angle at 60 cpm; (**b**) resistance change rate at 10 cpm; (**c**) comparison of voltages by angle at 30 cpm; (**d**) response time at bending angle of 90° and speed of 60 cpm; (**e**) voltage change with angle; (**f**) responsiveness with bending speed; (**g**) voltage and zoom graphs of the start and end cycles at bending angle of 90° and speed of 10 cpm.

Figure 3a shows the GF measured at a speed of 60 cpm at bending angles of 30°, 60°, and 90°, and presents the standard uncertainty of GF to evaluate the accuracy, reliability, consistency, and performance variability of the sensor. The standard uncertainty was calculated from the standard deviation obtained through repeated measurements, as shown in Equation (4) [30]. In this equation, u denotes the standard uncertainty, s is the standard deviation, and n represents the number of repeated measurements.

(4) $$u=\frac{s}{\sqrt{n}}$$

The GF and standard uncertainty according to the angle are as follows: 30° (GF = 123, standard uncertainty = ±0.4); 60° (GF = 271, standard uncertainty = ±3.2); 90° (GF = 698, standard uncertainty = ±7.8). GF tends to increase as the bending angle increases, and the standard deviation also increases. When the bending angle increases from 30° to 60°, the GF increases by about 2.2 times, and the standard uncertainty increases by about 8.3 times. When the bending angle increases from 60° to 90°, the GF increases by about 2.6 times, and the standard uncertainty increases by about 2.4 times. Figure 3b shows the resistance change rate for three strains (10%, 20%, and 30%). The resistance change rate and standard uncertainty are as follows: 10% strain (resistance change rate = 17.973, standard uncertainty = ±0.5); 20% strain (resistance change rate = 71.478, standard uncertainty = ±1.5); 30% strain (resistance change rate = 135.799, standard uncertainty = ±2.1). As the strain increases, the resistance change rate also increases and the standard uncertainty also increases proportionally. As a result, the sensor shows very stable and reliable performance at 30° and 10% strain. It shows good performance at 60° and 20% strain, but the standard uncertainty increases. It shows high GF at 90° and 30% strain, but the standard uncertainty increases. The increase in standard uncertainty with increasing bending angle and strain is expected to be due to the increased sensor sensitivity rather than a sensor performance issue. As shown in Figure 3c, the voltage value increased as the bending cycle increased at the three angles. However, when the average of the bending values for each angle was calculated and compared, 30° and 60° exhibited a difference of approximately 59%, and 60° and 90° exhibited a difference of approximately 19%, confirming the difference in voltage for each angle. As a result, the differences in the GF, resistance change rate, and voltage depending on the bending angle demonstrate that the developed knitted strain sensor exhibits high sensitivity and detects small changes.

In terms of responsiveness, the response speed of the strain sensor to bending motion and the time required by the sensor to return to its initial state were analyzed. A speed of 60 cpm, at which the sensor generally required approximately 1 s to return to its initial state from a bending motion, was considered suitable for checking real-time responsiveness and was considered as a reference. As shown in Figure 3d, the developed knitted strain sensor requires 0.5 s for the bending motion and 0.5 s for the unfolding motion, requiring 1 s to return to its initial state. These results confirm the fast responsiveness of the developed knitted strain sensor for performing both bending and unfolding motions in a short period of 1 s, which is a single cycle. Figure 3e shows the voltage changes when bent at angles of 30°, 60°, and 90° at a speed of 60 cpm. When bent at 30° and 60°, an error of about 0.1 s occurred, and there was no error at 90°, but a value similar to the maximum bending value was shown after about 0.1 s in all cycles. Figure 3f shows the voltage change when bent at 90° at speeds of 10 cpm, 30 cpm, and 60 cpm and demonstrates the responsiveness of the sensor according to the bending speed. A constant voltage value was observed even at different bending speeds, which means that the sensor can monitor the joint in real time regardless of the bending speed.

To clearly study the reproducibility, an additional 500 repeated bending experiments were performed. The reproducibility was measured as voltage peak-to-peak (Vp-p) uniformity and signal size over 500 bending cycles. These parameters are important for evaluating the electrical and physical performance of knitted strain sensors. The uniformity of Vp-p within the same cycle was also evaluated. A uniform baseline indicates that a knitted strain sensor exhibits excellent resilience and reproducibility during the bending and unfolding motion. The developed knitted strain sensor exhibits a pattern in which the voltage decreases during the bending motion and then increases during the unfolding motion. As shown in Figure 3g, the overall baseline exhibited a gentle shape even after 500 repeated bending. To evaluate this trend in more detail, we zoomed in and compared five cycles from the beginning (approximately 0–30 s), the middle (approximately 1500–1530 s), and the end (approximately 3000–3030 s) and observed the recovery process during the five cycles. In addition, a voltage change of about 0.3 V in the recovery state was confirmed due to hysteresis when the sensor returned to the extended state after 500 repeated bending. This problem can be improved by obtaining accurate measurements using algorithms that reduce errors due to hysteresis [31,32,33,34]. Alternatively, there is a method of returning a sensor value to an initial value by converting a baseline according to a voltage change [35]. The baseline converting methods can ensure long-term accuracy by calibrating zero-point drift that occurs after long-term use of sensors or environmental changes. Finally, there is a way to use highly elastic and excellent resilient materials when fabricating sensors [36,37,38]. This method can minimize friction between conductive yarn and non-conductive yarn and reduces hysteresis, which can alleviate the voltage increase problem in advance. The knitted strain sensor developed in this study demonstrated consistent patterns and stable performance during repetitive bending motions without additional compensation, demonstrating its potential for use in elbow joint monitoring.

In this study, to monitor elbow joints during dumbbell exercise, a knitted strain sensor with a plain stitch structure with a sensor length of 20 × 100 mm was fabricated using a computerized flat knitting machine. To examine whether the developed sensor can distinguish bending and unfolding motions and elbow joint angles in real time during exercise, a repeated bending experiment was conducted. As a result, the sensitivity of the developed knitted strain sensor was confirmed to be GF 111 at 30° and GF 698 at 90°. As the angle and deformation increased, the change in GF and resistance of the sensor increased and the standard uncertainty increased proportionally. Additionally, the difference in voltage value according to angle shows that the elbow angle can be detected during dumbbell movements. The responsiveness of the developed sensor was evaluated based on a speed of 60 cpm, at which the sensor needed approximately 1 s to complete each bending and unfolding motion (0.5 s each). We also investigated the effect of bending angle and velocity on the sensor’s sensing performance. An error of less than one second was observed depending on the bending angle, and it maintained a constant voltage value at various speeds. Therefore, the sensor has a fast response speed and allows real-time joint monitoring. To demonstrate the reproducibility of the sensor, we performed 500 cycles of additional experiments. All baselines showed a slight increase (approximately 0.3 V) when comparing the first and end cycles, but there was no problem in monitoring the joints during dumbbell motion. In addition, the baseline patterns remained smooth and regular. This shows that the sensor has excellent long-term stability and durability. To verify the performance of the developed sensor, it was compared with a recently reported textile-based strain sensor. As a result, the knitted strain sensor developed in this study showed high sensitivity, fast responsiveness, and excellent reproducibility. In particular, the detection performance was very stable even at low strains [39,40,41,42].

### 3.2. Applications of Knitted Strain Sensors

**Figure 4:**
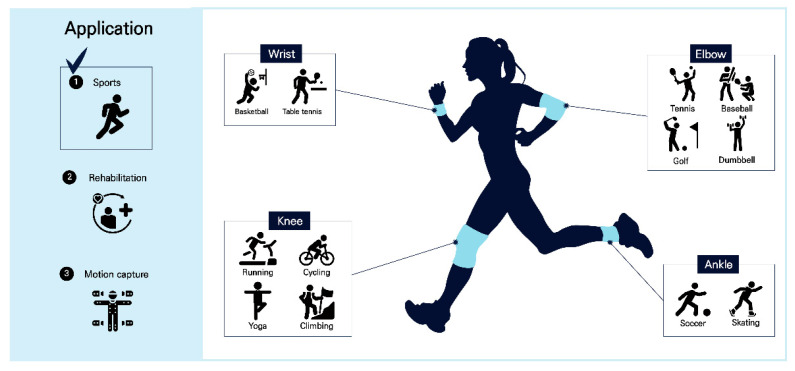
Possibility of utilizing knit strain sensors in wearable applications.

In this study, the developed sensor is primarily focused on elbow joint monitoring-based dumbbell exercise; however, in addition to dumbbell exercise, the sensor can also be used in sports that require joint monitoring, such as weight training, baseball, golf, and tennis. The fabricated sensor can also be used to monitor various bendable joints, such as knees, wrists, and ankles, not only elbows. In addition to sports, the sensor can be used in medical and rehabilitation fields, where accurate angles and postures are important, and in the entertainment field, which involves virtual reality and motion capture. The fabricated knitted strain sensor is made of a knitted material; thus, it is flexible, has a similar fit to regular clothing or protective gear, is in close contact with the skin, and exhibits excellent elasticity and wearability. Therefore, the fabricated sensor has the advantages of less discomfort or foreign body sensation, good breathability, high sensor sensitivity, fast responsiveness, excellent reproducibility, and being mass producible. The sensor is expected to be used as a wearable device in various fields. Figure 4 shows representative applications of knitted strain sensors mounted on different body parts in various sports.

## 4. Conclusions

In this study, a highly sensitive knitted strain sensor was fabricated using a silver-coated conductive yarn for monitoring elbow joint motions during dumbbell exercise. The sample has a plain stitch structure, which is a general knit structure. To improve the sensitivity, stability, and responsiveness of the developed sensor, the sensor part was designed to have a non-conductive yarn on the front and a conductive yarn on the back. To evaluate the electrical and physical characteristics of the developed sensor, repeated bending experiments were conducted at three speeds and angles, and the experimental results were evaluated in terms of sensitivity, responsiveness, and reproducibility. The developed sensor had high sensitivity (GF = 698) and GF increased with angle. The resistance change rate increased with strain, and there was a difference in the bending voltage value by angle. In addition, the sensor could detect bending and unfolding motion for 1 s and detected a constant voltage at various bending angles and speeds. The baseline of the voltage graph showed a regular pattern even after 500 repeated bending motions, confirming stable performance even during long-term repeated bending motion. These results show that the developed knitted strain sensor has high sensitivity, fast responsiveness, and excellent reproducibility, and that it has the potential for real-time joint monitoring.

Future research should focus on the practical applicability of the developed sensor. To monitor not only the elbow but also the knees, fingers, neck, and waist, the developed sensor should be directly applied to clothing and protective gear, and in-depth research on the detection performance and safety of movement of the sensor when worn is required. In addition, in order to monitor joints in the sports field, various environmental factors in daily life, such as humidity and temperature, sweat, and factors that can reduce the performance of the sensor, such as cleanability and durability, should be considered. This study contributes to the development of knitted strain sensors directly embedded in clothing and protective gear in various industrial fields that require joint monitoring.

## Data Availability

Data are contained within the article.
