# Correction: Choi, H.-J.; Kim, Y.-H. Development of Knitted Strain Sensor Optimized for Dumbbell Exercise and Evaluation of Its Electrical Characteristics. *Sensors* 2025, *25*, 3685

**DOI:** 10.3390/s26051424

**Published:** 2026-02-25

**Authors:** Hee-Ji Choi, Youn-Hee Kim

**Affiliations:** Department of Convergence Design and Technology, Kookmin University, Seoul 02707, Republic of Korea; hichio1228@kookmin.ac.kr

## Text Correction

1. There was an error in the original publication [1]. A correction has been made to the Abstract to prevent any potential misunderstanding and to clearly position the work with respect to the existing literature.

With growing interest in wearable technologies, the development of flexible sensors and products that can monitor the human body while being comfortable to wear is gaining momentum. While various textile-based strain sensors have been proposed, their implementation in practical, exercise-specific applications remains limited. In this study, we developed a knitted strain sensor that monitors elbow angles, focusing on dumbbell exercise, which is a basic exercise in sports, and verified its performance. The material of the developed knitted strain sensor with a plain stitch structure comprised a silver-coated nylon conductive yarn and an acrylic/wool blended yarn. To evaluate the electrical and physical characteristics of the developed sensor, a textile folding tester was used to conduct 100 repeated bending experiments at three angles of 30°, 60°, and 90° and speeds of 10, 30, and 60 cpm. The system demonstrated excellent elasticity, high sensitivity (gauge factor = 698), fast responsiveness, and reliable performance under repeated stress, indicating its potential for integration into wearable fitness or rehabilitation platforms. This study does not propose a new knitted sensor architecture; rather, it demonstrates an application-specific optimization and validation analysis of an established plated knitted structure for real-time elbow-joint monitoring during dumbbell exercise.

2. The following sentences have been added at the end of Section 1, Paragraph 1:

While numerous studies have explored knitted strain sensors using various loop topologies and stitch configurations, the present work does not aim to introduce a new knitted structure. Instead, it focuses on tailoring an established plated knitted configuration to a specific exercise scenario and experimentally validating its performance under application-relevant conditions.

3. In Section 1, Paragraph 5 has been updated to the following:

In this study, we utilized an established plated knitted structure and tailored it specifically for real-time elbow-joint monitoring during dumbbell exercise, one of the most fundamental strength-training movements. The sensor configuration was optimized for this application, and its electrical and mechanical performance was systematically evaluated under exercise-relevant conditions. The optimized sensor had high sensitivity and was able to stably detect long-term repeated sensing based on excellent reproducibility, and it showed fast responsiveness to deformation. In addition, the system’s integration of sensors, a microcontroller unit (MCU), lead wires, and a spring snap button provides measurement accuracy and a fast response time, as well as wireless operation, making it convenient to use. These wireless systems are capable of real-time elbow-joint monitoring and demonstrate practical applicability in wearable applications.

4. In Section 2, Subsection 2.1, Paragraph 1, the content “Stavrakis et al. [21] examined Aman’s silver-coated nylon conductive yarn to analyze the electrical properties of commercially available conductive yarns, and similar to the findings of this study, and found a significant discrepancy between the manufacturer’s specified resistance values and the actual measurements.” has been updated to “Stavrakis et al. [21] examined Aman’s silver-coated nylon conductive yarn to analyze the electrical properties of commercially available conductive yarns and reported a significant discrepancy between the manufacturer’s specified resistance values and the experimentally measured values, which is consistent with the findings of this study.”

5. In Section 2, Subsection 2.1, Paragraph 2, the content “Figure 1a,b show diagrams of a knitted strain sensor with a plain stitch structure comprising both conductive and non-conductive yarns and the appearance of the sensor when pulled in the front, back, and wale directions. Figure 1a shows the most common plain stitch structure with the sensor part knitted with only 1-ply of a conductive yarn; thus, the conductive yarn appears on the front, back, and both sides. Figure 1b shows a plain stitch structure designed to use two needles on the sensor part to position 1-ply of a non-conductive yarn on the front and 1-ply of a conductive yarn on the back. The sensor part of the sample comprised 2-ply, including 1-ply of a silver-coated conductive yarn and 1-ply of a non-conductive yarn from C&TEX (Seoul, Republic of Korea) at a 1:1 ratio of acrylic to wool, and the rest were made into a plain stitch structure with excellent elasticity using only 1-ply of a non-conductive yarn [25]. The overall length of the fabricated sample is 265 mm, the width is 90 mm, and the sensor part is 100 mm in length (88 wales) and 20 mm in width (14 courses).” has been updated to “Figure 1a,b show diagrams of a knitted strain sensor with a plain stitch structure comprising both conductive and non-conductive yarns and the appearance of the sensor when pulled in the front, back, and wale directions. Figure 1a shows the most common plain stitch structure with the sensor part knitted with only 1-ply of a conductive yarn; thus, the conductive yarn appears on the front, back, and both sides. Figure 1b shows a plain stitch structure designed to use two needles on the sensor part to position 1-ply of a non-conductive yarn on the front and 1-ply of a conductive yarn on the back. The sensor part of the sample comprised 2-ply, including 1-ply of a silver-coated conductive yarn and 1-ply of a non-conductive yarn from C&TEX (Seoul, Republic of Korea) at a 1:1 ratio of acrylic to wool, and the rest were made into a plain stitch structure with excellent elasticity using only 1-ply of a non-conductive yarn [25]. The overall length of the fabricated sample is 265 mm, the width is 90 mm, and the sensor part is 100 mm in length (88 courses) and 20 mm in width (14 wales).”

6. In Section 3, Subsection 3.1, Paragraph 6, the content “As shown in Figure 3d, the developed knitted strain sensor requires 0.5 s for the bending motion and 0.5 s for the unfolding motion, requiring 1 s to return to its initial state. These results confirm the fast responsiveness of the developed knitted strain sensor for performing both bending and unfolding motions in a short period of 1 s, which is a single cycle.” has been updated to “As shown in Figure 3d, the developed knitted strain sensor requires 0.5 s for the bending motion and 0.5 s for the releasing motion, requiring 1 s to return to its initial state. These results confirm the fast responsiveness of the developed knitted strain sensor for performing both the bending and releasing motions in a short period of 1 s, which is a single cycle.”

7. In Section 3, Subjection 3.1, Paragraph 7, the content “A uniform baseline indicates that a knitted strain sensor exhibits excellent resilience and reproducibility during the bending and unfolding motion. The developed knitted strain sensor exhibits a pattern in which the voltage decreases during the bending motion and then increases during the unfolding motion. As shown in Figure 3g, the overall baseline exhibited a gentle shape even after 500 repeated bending. To evaluate this trend in more detail, we zoomed in and compared five cycles from the beginning (approximately 0–30 s), the middle (approximately 1500–1530 s), and the end (approximately 3000–3030 s) and observed the recovery process during the five cycles.” has been updated to “A uniform baseline indicates that a knitted strain sensor exhibits excellent resilience and reproducibility during the bending and releasing motion. The developed knitted strain sensor exhibits a pattern in which the voltage decreases during the bending motion and then increases during the releasing motion. As shown in Figure 3g, the overall baseline exhibited a gentle shape even after 500 repeated bending. To evaluate this trend in more detail, we zoomed in and compared five cycles from the beginning (1–5 cycles), the middle (243–247 cycles), and the end (496–500 cycles) and observed the recovery process during the five cycles.”

8. In Section 3, Subsection 3.1, Paragraph 8, the content “To examine whether the developed sensor can distinguish bending and unfolding motions and elbow joint angles in real time during exercise, a repeated bending experiment was conducted.” has been updated to “To examine whether the developed sensor can distinguish the bending and releasing motions and elbow joint angles in real time during exercise, a repeated bending experiment was conducted.” The content “The responsiveness of the developed sensor was evaluated based on a speed of 60 cpm, at which the sensor needed approximately 1 s to complete each bending and unfolding motion (0.5 s each).” has been updated to “The responsiveness of the developed sensor was evaluated based on a speed of 60 cpm, at which the sensor needed approximately 1 s to complete each of the bending and releasing motion (0.5 s each).”

9. In Section 4. The word “unfolding” has been updated to “releasing”.

## Figure Correction

In the original publication, there were mistakes in Figures 1 and 3 as published. The corrected versions of Figure 1 and Figure 3 are shown below.

## References Correction

There were typos in References 3, 11, 20 and 22. The corrected versions of References 3, 11, 20 and 22 are listed as follows:

3. Bozali, B.; Ghodrat, S.; Jansen, K.M. Design of Wearable Finger Sensors for Rehabilitation Applications. *Micromachines* **2023**, *14*, 710. https://doi.org/10.3390/mi14040710.
11. Seyedin, S.; Moradi, S.; Singh, C.; Razal, J.M. Continuous production of stretchable conductive multifilaments in kilometer scale enables facile knitting of wearable strain sensing textiles. *Appl. Mater. Today* **2018**, *11*, 255–263. https://doi.org/10.1016/j.apmt.2018.02.012.
20. Rumon, M.A.; Cay, G.; Ravichandran, V.; Altekreeti, A.; Gitelson-Kahn, A.; Constant, N.; Solanki, D.; Mankodiya, K. Textile knitted stretch sensors for wearable health monitoring: Design and performance evaluation. *Biosensors* **2023**, *13*, 34. https://doi.org/10.3390/bios13010034.
22. Chui, Y.; Yang, C.; Tong, J.; Zhao, Y.; Ho, C.; Li, L. A systematic method for stability assessment of Ag-coated nylon yarn. *Text. Res. J.* **2015**, *86*, 787–802. https://doi.org/10.1177/0040517515595032.

Two Master’s theses sources have been replaced with two peer reviewed articles. The new References 27 and 28 are listed as:

27. Oh, Y.-K.; Kim, Y.-H. Evaluation of Electrical Characteristics of Weft-Knitted Strain Sensors for Joint Motion Monitoring: Focus on Plating Stitch Structure. *Sensors* **2024**, *24*, 7581. https://doi.org/10.3390/s24237581.
28. Amjadi, M.; Kyung, K.U.; Park, I.; Sun, M. Stretchable, Skin-Mountable, and Wearable Strain Sensors and Their Potential Applications: A Review. *Adv. Funct. Mater.* **2016**, *26*, 1678–1698. https://doi.org/10.1002/adfm.201504755.

The authors state that the scientific conclusions are unaffected. This correction was approved by the Academic Editor. The original publication has also been updated.

## Figures and Tables

**Figure 1:**
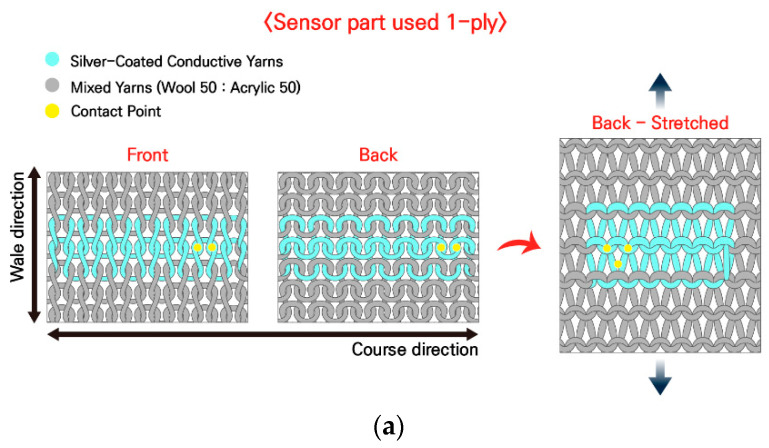

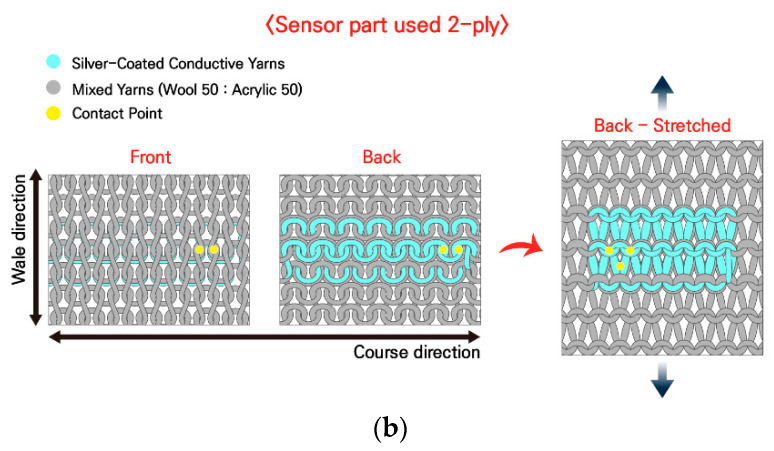
Knitted strain sensor. (**a**) Schematic of the 1-ply plain stitch; (**b**) schematic of the 2-ply plain stitch.

**Figure 3:**
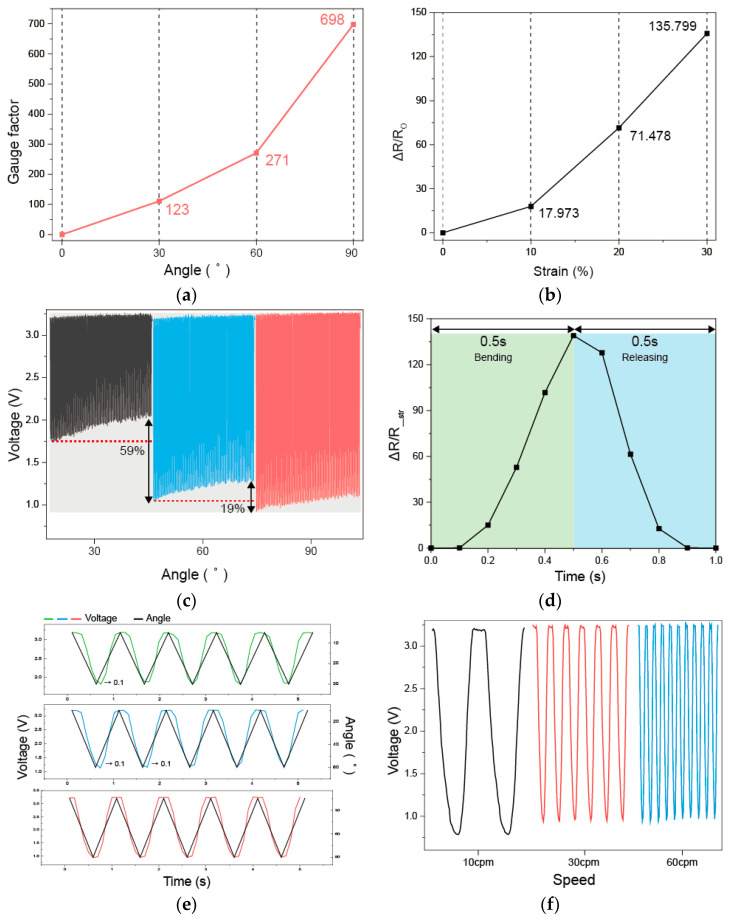

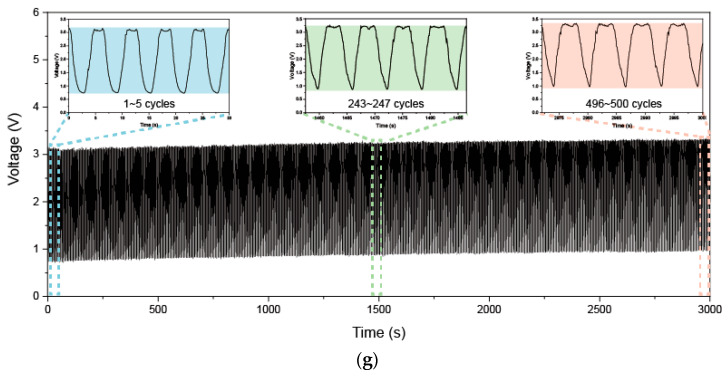
Results of repeated bending tests: (**a**) GF by angle at 60 cpm; (**b**) resistance change rate at 10 cpm; (**c**) comparison of voltages by angle at 30 cpm; (**d**) response time at a bending angle of 90° and a speed of 60 cpm; (**e**) voltage change with angle; (**f**) responsiveness with bending speed; (**g**) voltage and zoom graphs of the start and end cycles at a bending angle of 90° and a speed of 10 cpm.
